# Efficacy of Minocycline Hydrochloride Aspiration Sclerotherapy for Symptomatic Simple Hepatic Cysts: Does Clinical Outcome Vary With the Number of Injections?

**DOI:** 10.7759/cureus.100090

**Published:** 2025-12-25

**Authors:** Tetsushi Azami, Yuichi Takano, Naoki Tamai, Jun Noda, Fumitaka Niiya, Masatsugu Nagahama

**Affiliations:** 1 Department of Gastroenterology, Showa Medical University Fujigaoka Hospital, Yokohama, JPN; 2 Division of Gastroenterology, Department of Internal Medicine, Showa Medical University Fujigaoka Hospital, Yokohama, JPN

**Keywords:** aspiration sclerotherapy, hepatic cyst, liver cyst drainage, minocycline hydrochloride, simple hepatic cyst

## Abstract

Background and aim

Minocycline hydrochloride (MINO) aspiration sclerotherapy has been reported as a therapeutic strategy for symptomatic simple hepatic cysts. However, treatment methods vary between studies, and the optimal frequency of MINO administration remains debated. This study aimed to compare the efficacy and safety of single-dose versus multiple-dose MINO aspiration sclerotherapy for symptomatic simple hepatic cysts, with clearly defined primary and secondary clinical outcomes.

Methods

This was a single-center retrospective study. Patients who underwent MINO aspiration sclerotherapy for symptomatic simple hepatic cysts at Fujigaoka Hospital from January 2017 to March 2023 were included. Participants were categorized into single- and multiple-dose MINO groups for comparison. Before admission, patients were informed of their respective treatment schedules by their attending physicians, and the number of MINO doses administered was determined as either a single or multiple doses.

Results

The single- and multiple-dose groups comprised five and eight cases, respectively. Clinical success rates were 80% (4/5) and 87.5% (7/8) in the single- and multiple-dose groups, respectively. The median cyst volume reduction rate was 96.5% (range, 93.0-99.1%) and 99.1% (range, 80.7-99.8%) in the single- and multiple-dose groups, respectively. Symptom disappearance rates were 80% (4/5) and 100% (8/8) in the single- and multiple-dose groups, respectively, with no significant difference. Adverse event (AE) rates were 60% (3/5) and 62.5% (5/8) in the single- and multiple-dose groups, respectively, with no statistically significant difference detected. The single-dose group tended to have a shorter median length of hospital stay (18 vs. 23 days); however, this difference was not significant.

Conclusions

Although the sample size was small, single-dose minocycline therapy showed outcomes that were not clearly inferior to those of multiple-dose treatment. It may also reduce AEs and shorten hospitalization, but larger studies are needed to confirm these findings. A single-dose regimen could be considered a treatment option in selected patients.

## Introduction

Simple hepatic cysts are considered congenital in origin and are characterized by a lining of epithelial cells [1]. They have a prevalence of approximately 18% [2-4] and are frequently encountered in routine clinical practice; however, the majority of patients are asymptomatic and require no treatment.

Conversely, approximately 15% of patients with simple hepatic cysts are symptomatic [5]. Symptoms are varied, with abdominal tightness being the most common. Although rare, other symptoms, including obstructive jaundice, pancreatitis, and edema due to venous compression, have also been reported [6,7]. Volume-reducing therapies are generally indicated for patients with symptomatic simple hepatic cysts [8]. Surgery (cystotomy or hepatectomy) is the definitive treatment; however, aspiration sclerotherapy, which is less invasive, is often selected based on operative tolerance and patient preference. In addition to percutaneous therapy, endoscopic ultrasonography-guided therapy has recently been reported [9,10].

Multiple agents have been employed in percutaneous aspiration sclerotherapy. Among them, ethanol sclerotherapy has been widely reported, whereas minocycline hydrochloride (MINO) has been occasionally used due to the risk of adverse events (AEs), including ethanol intoxication. Despite scattered reports on percutaneous aspiration sclerotherapy using MINO, treatment protocols vary considerably across studies, and the optimal frequency and concentration of MINO administration remain debated [11-17]. The number of MINO administrations may influence treatment invasiveness, procedure-related AEs such as pain, and length of hospital stay, thereby affecting both patient burden and healthcare resource utilization. From a clinical perspective, identifying the minimum effective number of MINO administrations is therefore an important issue.

Accordingly, this study aimed to compare the efficacy and safety of percutaneous aspiration sclerotherapy with MINO between patients treated with a single dose and those treated with multiple doses. The primary outcomes were clinical success, defined as a ≥50% reduction in cyst volume maintained at one year after treatment, and symptom disappearance, defined as resolution at six months maintained up to one year. Secondary outcomes included cyst volume reduction rate at one year, technical success, AEs, and length of hospital stay.

## Materials and methods

Study design and participants 

This was a single-center retrospective observational study. Patients who underwent MINO aspiration sclerotherapy for symptomatic simple hepatic cysts at Showa University Fujigaoka Hospital from January 1, 2017, to March 31, 2023, were included. The follow-up period was one year, and patients treated for less than one year were excluded.

MINO aspiration sclerotherapy exerts its therapeutic effect through chemical injury to the hepatic cyst epithelium. However, active infection can induce persistent alterations in intracystic pH and inflammatory activity, which may interfere with the efficacy of this treatment. In addition, infectious hepatic cysts are often accompanied by systemic illness requiring antimicrobial therapy and prolonged hospitalization, which can substantially influence the patient’s general condition, AE profile, and length of hospital stay independently of the sclerotherapy itself. For these reasons, cases of infectious hepatic cysts were excluded. Cysts were defined as infected when cultures of aspirated cyst fluid yielded positive results.

Patient and cyst characteristics were retrospectively reviewed using medical records. Original data were accessed for research purposes between October 30, 2024, and December 4, 2024. This study was conducted in accordance with the Declaration of Helsinki and was approved by the Showa University Research Ethics Review Board (approval number 2024-113-B; registration date October 29, 2024; retrospectively registered).

Outcomes and definitions

Outcomes were compared between the single- and multiple-dose MINO groups.

**Figure 1:**
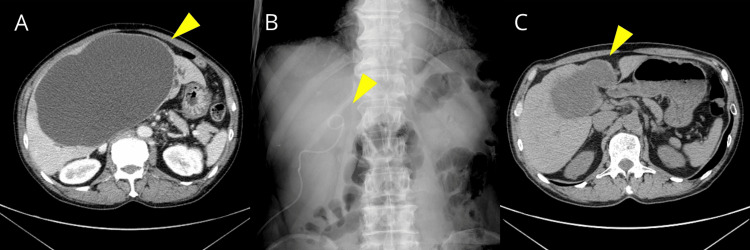
Example of clinical response (A) Axial abdominal CT: Image showing a simple hepatic cyst prior to percutaneous aspiration sclerotherapy. (B) Fluoroscopic image: Image showing placement of a pigtail catheter and instillation of MINO during the sclerotherapy procedure. (C) Axial abdominal CT: Image obtained one year after treatment, demonstrating a reduction in cyst volume to less than 50%. MINO, minocycline hydrochloride

The primary outcome measures were clinical success and symptom disappearance. Clinical success was defined as a ≥50% reduction in cyst volume following treatment, maintained for one year after MINO administration. Symptom disappearance was defined as the resolution of symptoms at six months following MINO treatment, maintained for up to one year (Figure 1).

CT or MRI was used to measure cyst volume, calculated assuming the cyst to be an ellipsoid using the following formula:

$\text{Cyst volume (mL)} = \text{height (cm)} \times \text{width (cm)} \times \text{length (cm)} \times 0.523$

Secondary outcomes included cyst volume reduction rate, technical success rate, AE rate, and length of hospital stay. The cyst volume reduction rate was calculated at one year after MINO administration; patients requiring retreatment were excluded from this analysis. AEs were evaluated separately during catheter insertion and MINO administration, and severity was assessed using the Clavien-Dindo classification [18]. Pain was specifically assessed using the Numerical Rating Scale (NRS) at the time of catheter insertion and MINO administration [19]. The AE rate was determined per session, defined as the period from catheter insertion to MINO injection and catheter removal. Hospital stay was defined as the period from the first catheter placement to patient discharge.

Procedure details

Abdominal ultrasonography and contrast-enhanced CT or MRI were used to diagnose simple hepatic cysts. Before admission, the attending physician explained the procedure, potential AEs, and the anticipated length of hospital stay to the patient. The number of MINO administrations was determined according to the physician’s clinical judgment and patient preference, based on overall clinical assessment, rather than through randomization.

When no allergic reactions were observed, analgesic medication (pentazocine 15 mg) was administered immediately before the procedure. Following local anesthesia with lidocaine, an 18-G ultrasound needle (disposable puncture needle; Hanaco Medical, Saitama, Japan) was inserted under ultrasound guidance. A 0.035-inch guide wire (Radifocus; Terumo, Tokyo, Japan) was then placed in the hepatic cyst under fluoroscopic guidance. After making a small skin incision and dilating it with a dilator (JCD dilator; Cook Medical, Bloomington, Indiana, USA), either a 6- or 8-Fr pigtail catheter (Chole-Cath; Uresil LLC, Skokie, Illinois, USA) was inserted.

The drainage fluid underwent multiple cytological examinations to inspect for malignant cells. Several days later, analysis of bilirubin levels in the cystic fluid and drain contrast injection confirmed no biliary system involvement. The MINO dose was selected based on previously published reports [11-17], which used doses ranging from 100 mg to 600 mg. A dose of 500 mg was therefore chosen for the present study.

After confirming via CT that the catheter was properly placed in the cyst, MINO 500 mg with 50 mL of saline was injected, and the catheter was clamped. In the single-dose group, the catheter was removed either the following day or a few days later. In the multiple-dose group, the catheter was reopened, and the injection of MINO 500 mg with 50 mL of saline was repeated every few days until the drainage volume significantly decreased or CT revealed no cyst growth. Patients were followed as outpatients after completion of puncture sclerotherapy. CT or MRI was routinely performed one year after treatment, with additional imaging obtained earlier if subjective symptoms arose. All images were evaluated by a gastroenterologist.

Statistical analysis

Continuous variables are presented as medians (ranges) and categorical variables as percentages. Outcomes between the single- and multiple-dose groups were compared using the Mann-Whitney U test for continuous variables and Fisher’s exact test for categorical variables. A sensitivity analysis was performed by excluding one patient who received eight MINO administrations to assess the robustness of the results. P < 0.05 was considered statistically significant. Data analyses were performed using R version 4.0.3 (R Foundation for Statistical Computing, Vienna, Austria).

## Results

Participants’ clinical characteristics 

**Figure 2:**
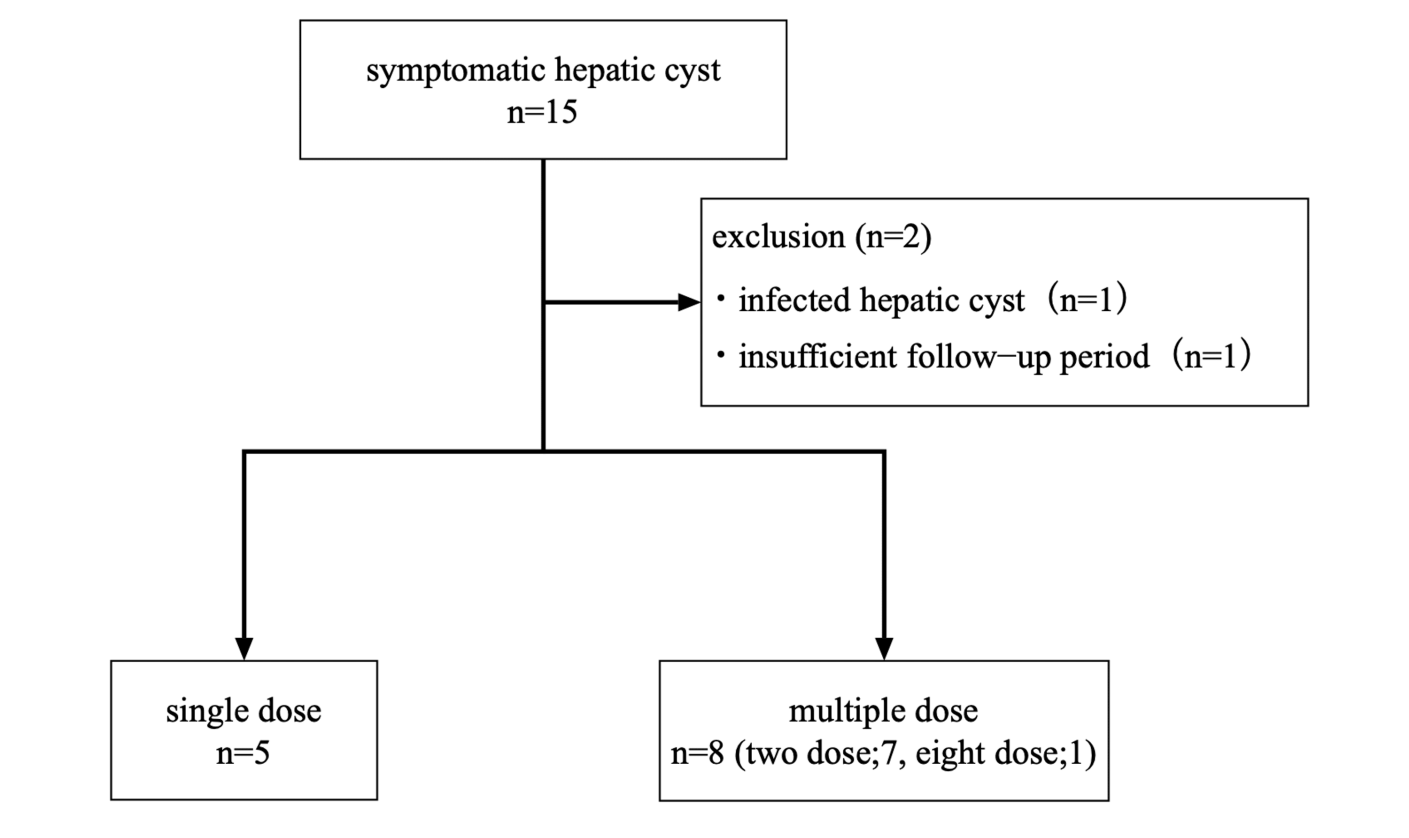
Flowchart of participant enrollment

Fifteen patients with symptomatic hepatic cysts underwent percutaneous aspiration sclerotherapy from January 1, 2017, to March 31, 2023. One patient was excluded due to an infectious hepatic cyst, and another was excluded for follow-up of less than one year. Ultimately, five patients were included in the single-dose group and eight patients in the multiple-dose group (seven patients received two doses, and one patient received eight doses) (Figure 2).

In the patient who received eight doses of MINO, drainage did not decrease even after repeated administration. This case was considered a clinical failure, and discontinuation of treatment was discussed; however, therapy continued at the patient’s request. As a result, the patient received eight doses of MINO, representing an outlier. Nevertheless, the patient adhered to the treatment protocol, did not meet any exclusion criteria, and was therefore included in the study.

The participants’ characteristics are presented in Table 1. The median age was 75 years (range, 15-84) in the single-dose group and 73.5 years (range, 63-92) in the multiple-dose group, with no significant differences. In the single-dose group, only one patient was male; the remaining patients were female. No significant differences were observed in the Charlson Comorbidity Index (CCI). The median CT values were 12.1 Hounsfield units (HU) (range, 5.6-20) and 14.8 HU (range, 8.36-25.9) in the single- and multiple-dose groups, respectively, with no statistically significant difference (Table 1). The mean CT values in cases of clinical failure were 19.8 HU and 20.0 HU. Drainage fluid was submitted for cytology, and none of the patients showed evidence of malignancy. 

**Table 1:** Comparison of participants’ characteristics Continuous variables were analyzed using the Mann-Whitney U test, and categorical variables were evaluated using Fisher’s exact test, as appropriate. Categorical variables are presented as n (%), and continuous variables as median (range). CCI, Charlson Comorbidity Index; IVC, inferior vena cava

Characteristics	Overall (n = 13)	Single dose (n = 5)	Multiple dose (n = 8)	Test statistic	p-Value
Age (years), median (range)	74 (15-92)	75 (15-84)	73.5 (63-92)	U = 16.5	0.66
Sex (male), n (%)	1 (7.7)	1 (20)	0	Fisher: -	0.385
CCI, median (range)	5 (0-9)	5 (0-6)	4 (3-9)	U = 20.5	0.433
History of antithrombotic medication, n (%)	2 (15.4)	2 (40)	0	Fisher: -	0.128
Cyst location in right liver lobe, n (%)	8 (61.5)	3 (60)	5 (62.5)	χ² = 0	1
Baseline cyst volume (mL), median (range)	1083 (176-4041)	1002 (176-2958)	1117 (526-4041)	U = 15	0.524
History of intracystic hemorrhage, n (%)	3 (23.1)	0	3 (37.5)	Fisher: -	0.231
Symptoms, n (%)					
Abdominal pain	9 (69.2)	2 (40)	7 (87.5)	Fisher: -	0.217
Cholangitis	1 (7.7)	1 (20)	0	Fisher: -	0.385
Cholecystitis	1 (7.7)	1 (20)	0	Fisher: -	0.385
Pancreatitis due to pancreatic duct stenosis	1 (7.7)	1 (20)	0	Fisher: -	0.385
Edema due to IVC pressure	1 (7.7)	0	1 (12.5)	Fisher: -	1

Outcomes

The clinical success rates were 80% (n = 4) and 87.5% (n = 7) in the single- and multiple-dose groups, respectively, with no significant difference. Symptom disappearance rates were 80% (n = 4) and 100% (n = 8) in the single- and multiple-dose groups, respectively, with no statistically significant difference. Both groups achieved a 100% technical success rate.

At one year after treatment, the median cyst volume was 22.5 mL (range, 5-183 mL) in the single-dose group and 30 mL (range, 1-204 mL) in the multiple-dose group, with an overall median volume of 30 mL (range, 1-204 mL). The median cyst volume reduction rate was 96.5% (range, 93.0-99.1%) in the single-dose group and 99.1% (range, 80.7-99.8%) in the multiple-dose group, with an overall median reduction rate of 97.4% (range, 80.7-99.8%). No statistically significant differences were observed.

The AE rates were 60% (grade I pain in two cases; grade I temperature increase in one case) and 62.5% (grade I pain in three cases; grade I temperature increase in two cases) in the single- and multiple-dose groups, respectively, with no statistically significant difference. The median NRS for pain among affected patients was 7/10 (range, 6-8). Patients who experienced pain or elevated body temperature during the first MINO administration exhibited similar symptoms during subsequent administrations. In the multiple-dose group, these symptoms persisted consistently across each administration.

The median length of hospital stay was 18 days (range, 8-23) in the single-dose group and 23 days (range, 18-71) in the multiple-dose group, with no statistically significant difference; however, the single-dose group tended to have a shorter stay (Table 2).

**Table 2:** Summary of outcomes Continuous variables were compared using the Mann-Whitney U test, and categorical variables were assessed using Fisher’s exact test. Categorical data are presented as n (%), and continuous variables as median (range). AE, adverse event

Outcomes	Overall (n = 13)	Single dose (n = 5)	Multiple dose (n = 8)	Test statistic	p-Value
Primary outcomes					
Clinical success rate, n (%)	11 (84.6)	4 (80)	7 (87.5)	Fisher: -	1
Symptom disappearance rate, n (%)	12 (92.3)	4 (80)	8 (100)	Fisher: -	0.385
Secondary outcomes					
Volume reduction rate (%), median (range)	97.4 (80.7-99.8)	96.5 (93-99.1)	99.1 (80.7-99.8)	U = 8.5	0.343
Technical success rate, n (%)	13 (100)	5 (100)	8 (100)	Fisher: -	1
AE rate, n (%)	8 (61.5)	3 (60)	5 (62.5)	Fisher: -	1
Length of hospital stay (days), median (range)	20 (8-71)	18 (8-23)	23 (18-71)	U = 8.5	0.108

In a sensitivity analysis excluding the patient who received eight MINO administrations, the results were consistent with the primary analysis.

## Discussion

The formation of simple hepatic cysts involves abnormal ductal plate maturation during fetal life. Owing to ductal plate malformation, the ductal plate separates from the bile duct, leading to fluid secretion by epithelial cells and subsequent cyst formation [1]. Percutaneous aspiration sclerotherapy aims to reliably shrink cysts by draining the cystic fluid and administering drugs that damage the epithelial cells, thereby reducing their secretory function. Reported sclerosing agents include 100% ethanol, minocycline (MINO), 20% sodium chloride, and polidocanol; however, no single agent has demonstrated clear superiority, and a standardized treatment protocol has yet to be established. A systematic review of percutaneous aspiration sclerotherapy using sclerosing agents such as ethanol or MINO reported cyst volume reduction in 76-100% of patients and symptom resolution in 56-100% [13]. In this study, 84.6% of patients in both groups combined achieved the primary endpoint of cyst volume reduction (clinical success), and 92.3% achieved symptom resolution, consistent with previous reports.

A mean CT value of 13.34 HU or higher has been reported as a predictor of poor response to MINO aspiration sclerotherapy [14]; in this study, no difference in mean CT values was observed between the single- and multiple-dose groups. Comparison of the two groups revealed no significant differences: the single-dose group had a clinical success rate of 80% (4/5 patients) and a symptom disappearance rate of 80% (4/5 patients), whereas the multiple-dose group had a clinical success rate of 87.5% (7/8 patients) and a symptom disappearance rate of 100% (8/8 patients). Similarly, cyst volume reduction rates at one year post-treatment were high in both groups, with no statistically significant differences, supporting consistency across analytical approaches.

Hahn et al. reported that short-term cyst enlargement following percutaneous aspiration sclerotherapy is a transient inflammatory reaction that typically resolves within a few months and requires no intervention [20]. The EASL Clinical Practice Guidelines recommend follow-up for six months after treatment even if the cyst increases in size [7]. Hahn et al. also noted that for cysts larger than 100 mL, the sclerosing drug should be administered at least twice during the same session [20]. Administering the drug after sufficient drainage may disrupt the epithelial cells, and cyst enlargement within days of treatment likely reflects an inflammatory response rather than the need for additional dosing.

No differences in age, CCI, or antithrombotic medication use were observed between the groups, and AE rates were similar (60% and 62.5% in the single- and multiple-dose groups, respectively). Although relatively high, all AEs were grade I (mild). Patients in the multiple-dose group who experienced pain or elevated body temperature after the first MINO administration exhibited similar symptoms with each subsequent dose.

In clinical practice, patients unable to eat due to fever or pain are frequently encountered. Even short-term fasting during hospitalization has been associated with muscle weakness, particularly in elderly patients. Therefore, although all MINO-related AEs in this study were mild, their potential clinical impact should not be overlooked [21]. Reducing the number of MINO administrations may therefore decrease cumulative AEs. Accordingly, while no statistically significant differences were observed between the groups, the single-dose strategy may offer the advantage of a lower cumulative AE burden.

The relatively high incidence of pain observed in this study warrants consideration. Previous protocols have mixed MINO with local anesthetics such as lidocaine or mepivacaine to reduce injection-related pain [10,11]. In this study, MINO was administered without a local anesthetic, which may partly explain the relatively high rate of pain-related AEs in both groups.

Although not statistically significant, the single-dose group tended to have a shorter hospital stay than the multiple-dose group. While the sample size precludes definitive conclusions, shorter hospital stays are clinically relevant for preserving activities of daily living and cognitive function, particularly in older adults, and for controlling healthcare costs. Prolonged hospital stays, including a maximum of 71 days in the multiple-dose group, were related to the treatment protocol rather than procedure-related complications. In the multiple-dose group, MINO administration continued until drainage volume decreased sufficiently or imaging confirmed no further cyst growth, contributing to extended hospitalization in certain cases. Sensitivity analysis excluding the patient who received eight MINO administrations did not alter the overall results or conclusions, indicating that this outlier did not unduly affect outcomes.

When interpreting hospitalization outcomes, cyst characteristics that independently affect the clinical course should be considered. Unlike infectious hepatic cysts, intracystic hemorrhage was not an exclusion criterion in this study. Among included patients, hemorrhagic findings had resolved clinically and radiologically before MINO administration, and no active infection was present at the time of treatment. Although blood components can transiently alter cyst contents and intracystic pH, these effects are typically self-limited, and their influence on MINO’s chemical injury mechanism and systemic condition was considered minimal compared with that of active infection.

This study has several limitations. First, the small sample size reduced statistical power, increasing the risk of Type II error. The absence of statistically significant differences between groups should not be interpreted as evidence of equivalence; rather, these findings are exploratory and hypothesis-generating. Second, selection bias is inherent, as treatment decisions were based on physician judgment and patient preference rather than random allocation. Although baseline characteristics were broadly comparable, residual confounding from unmeasured factors may have influenced treatment selection and outcomes. Third, while a MINO dose of 500 mg with 50 mL saline was used based on prior studies, further research is needed to explore factors beyond dosing frequency, including the optimal dose. Finally, as a single-center study conducted under institution-specific practices, the generalizability of findings may be limited.

## Conclusions

Despite the limited sample size, our results suggest that single-dose MINO therapy produced outcomes that were not clearly inferior to those of multiple-dose administration in this cohort. This approach may also reduce AEs and shorten hospitalization, although these findings should be interpreted cautiously and require validation in larger prospective studies. Taken together, a single-dose regimen may be considered a viable treatment option for selected patients with symptomatic simple hepatic cysts.
